# Patient experience and overall satisfaction after emergency abdominal surgery

**DOI:** 10.1186/s12893-017-0271-5

**Published:** 2017-07-01

**Authors:** C. H. Jones, S. O’Neill, K. A. McLean, S. J. Wigmore, E. M. Harrison

**Affiliations:** Clinical Surgery, University of Edinburgh, Royal Infirmary of Edinburgh, 51 Little France Crescent, Edinburgh, EH16 4SA UK

**Keywords:** Patient experience, Emergency surgery, Patient satisfaction

## Abstract

**Background:**

There is a growing recognition of the importance of patient experience in healthcare, however little is known in the context of emergency abdominal surgery. This study sought to quantify the association between patient experience and overall satisfaction.

**Methods:**

Patient demographics, operation details and 30-day clinical outcome data of consecutive patients undergoing emergency abdominal surgery were collected. Data was collected using validated Patient Reported Experience Measures (PREMs) questionnaires. Categorical data were tested using Mann Whitney U test. Multivariable regression was used to determine independent factors associated with satisfaction.

**Results:**

In a well-fitting multivariable analysis (R^2^ = 0.71), variables significantly associated with a higher global satisfaction score were “sufficient information given about treatment” (β = 0.86, 95% CI 0.01–1.70, *p* = 0.047), "sufficient explanation of risks and benefits of surgery" (β = 1.26, 95% CI 0.18–2.34, *p* = 0.020), “absence of night-time noise” (β = 1.35, 95% CI 0.56–2.14, *p* = 0.001) and “confidence and trust in nurses” (β = 1.51, 95% CI 0.54–2.49, *p* = 0.003).

**Conclusions:**

Overall patient satisfaction was strongly associated with perceptions of good communication and transfer of information. Confidence and trust in the clinical team is an important determinant of patient experience. Improving the ward environment by reducing noise at night may also improve the overall experience and satisfaction in emergency surgery.

## Background

There is increasing recognition that traditional indicators of clinical outcomes such as mortality and complication rates are inadequate surrogate measures for good care, and that a more holistic approach is needed [1]. In addition to its intrinsic ethical value, good patient experience has been consistently positively associated with patient safety and clinical effectiveness across a wide range of disease areas, study design settings, population groups and outcome measures [2]. Both the 2008 report “High Quality Care For All” [3] and the 2010 White Paper "Equity and excellence: liberating the NHS" [4] have enshrined good patient experience as a cornerstone of good clinical care and a central goal for the NHS.

In response to this, Patient Reported Experience Measures (PREMs) have been developed to quantify patient experience in order to inform broader quality improvement strategies [5]. However, the focus of PREM research conducted to date has either been generic or focused on chronic medical conditions. Questionnaires have been developed for general adult inpatients [6], general practice [7], children’s services [8], mental health [9] and maternity services [10] within the NHS. Condition specific tools have been developed for cancer [11], diabetes [12], coronary heart disease [13] and stroke [14]. Very little research has been done looking specifically at experiences of patients undergoing emergency surgery.

Emergency surgery can be a particularity challenging area for patient experience. Patients present acutely unwell, in pain and distressed. Admission by definition is unplanned and frequently occurs at night. The patient journey within the hospital can be complicated, with numerous transfers between clinical areas and teams making continuity of care challenging. In comparison with elective patients, care is often co-ordinated by more junior members of staff and patients may not have access to additional avenues of support such as specialist nurses.

The aim of this study was to explore the relationship between patient experience and overall satisfaction for patients undergoing emergency intra-abdominal surgery.

## Methods

### Study design

This study used an abbreviated version of the General Inpatient Survey (GIS), a validated instrument designed for use in the NHS [15, 16]. The GIS questionnaire is designed for elective and emergency patients and was modified for use in the purely emergency setting by removing questions specifically concerning elective patients. Ethical approval was sought from the South East Scotland Research Ethics Service (ref NR/1412AB6), and a waiver obtained to proceed without formal ethical review, as the project was limited to using data obtained as part of usual care relating to the evaluation of service delivery.

This study included sequential patients undergoing emergency intra-peritoneal surgery within a four-week study period, including those who had undergone intra-peritoneal gynaecological surgery, but excluding caesarean sections. ‘Emergency’ surgery was defined as unplanned, non-elective, same admission procedures. Elective and semi-elective patients were excluded. Verbal consent was obtained from each patient.

### Data collection

Eligible patients were identified prospectively. Demographic data was collected for all patients using theatre records, inpatient notes and electronic patient records over a four week study period in November 2014 at the Royal Infirmary of Edinburgh.

Patient experience questionnaires were administered either over the phone shortly after discharge or in person at the time of discharge and recorded in an institutional database.

### Data analysis

A continuous overall satisfaction score out of 10 for the whole inpatient experience was used in univariable analysis. Questions were structured using a three point Likert scale, with participants asked if they had experienced a particular variable at all times (1), sometimes (2) or not at all (3). If the question concerned a negative experience, for example noise at night, participants were asked if the had experienced that variable never (1), sometimes (2) or at all times (3). Responses combined into dichotomous categories for analytic purposes using a “top-box” approach, comparing the mean overall satisfaction score of those who had experienced a particular variable at all times (1), to that of those who had not (2–3) [17–19]. The statistical significance of the association between experiencing a variable at all times and the mean overall satisfaction score was tested using the Mann Whitney U test. Multivariable linear regression models were constructed using forward and backward manual variable selection strategies and included factors most significantly associated with satisfaction in univariate analysis. Model fit was judged using likelihood ratio tests/Akaike information criteria. No first-order interactions were identified and appropriate model diagnostics were checked (outliers/influential observations, normality of residuals, and heteroscedasticity). Data are presented as mean and standard deviation unless otherwise stated. All statistical analyses were conducted using RStudio v2.1 (R Foundation for Statistical Computing).

## Results

### Study recruitment

**Fig. 1:**
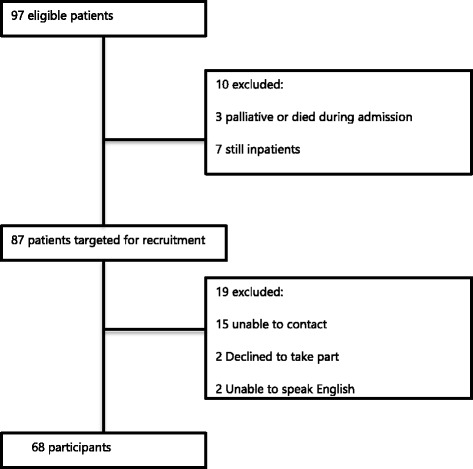
Flow diagram describing participant recruitment. Of 97 patients who underwent emergency intra-abdominal surgery within the 4-week study period, 10 were immediately excluded as they were either dead or were still inpatients at the end of the data collection period. Attempts were made to contact the remaining 87 patients, resulting in 68 participants and 19 non-participants. The main reason for non-recruitment was inability to contact the patients by telephone

97 patients met the inclusion criteria and were followed throughout their admission (Fig. 1). Those who had not been discharged by the end of the data collection period were excluded, giving a total of 87 eligible patients. Of those, 68 were recruited, giving a response rate of 78%. There was no significant difference between the participants and the non-participants (Table 1).

**Table 1:** A comparison of the demographics of the study participants with the other patients who met the inclusion criteria but did not participate. Data are *n* (%) unless otherwise stated

	Participants (*N* = 68)	Non-participants (*N* = 19)	*p*-value
Age (median, range)	42 (13–83)	30 (15–84)	0.990
Age (categorical)			0.267
Under 18	5	2	
18–65	50	13	
Over 65	13	4	
Gender			0.540
Male	18 (26)	4 (33)	
Female	50 (74)	15 (67)	
ASA			0.208
1	32 (47)	8 (42)	
2	19 (28)	3 (16)	
3+	6 (9)	5 (26)	
Unknown	11 (16)	3 (16)	
Average length of stay (days, range)	2.65 (1–18)	3.27 (1–11)	0.531
Operation			0.382
Cholecystectomy	24 (35)	8 (42)	
Appendectomy	24 (35)	3 (16)	
Ovarian/Salpingectomy	7 (10)	2 (10)	
Other	13 (20)	6 (32)	

### Univariate analysis

The mean overall satisfaction score was 8.2/10 (standard deviation (SD) = ±1.65). Variables encompassing different aspects of patient experience associated with a significantly higher mean satisfaction score are shown in Table 2.

**Table 2:** Analysis of the association between individual patient reported experience measures and overall patient-reported satisfaction, and multivariate linear regression of significant variables. Patient-reported satisfaction data are mean overall satisfaction score out of 10

	Patient-reported satisfaction					
	Univariable analysis			Multivariable linear regression		
	Experienced at all time (±SD)	Sometimes or never experienced (±SD)	*p*-value	β-estimate	95% CI	*p*-value
Admission						
Sufficient information in ED	8.46 (1.21)	7.35 (2.41)	0.220			
Sufficient privacy in the ED	8.22 (1.61)	6.33 (3.06)	0.220			
Sufficient information in the Hot Clinic	7.91 (1.56)	8.66 (1.21)	0.440			
Did not experience a long wait for bed in ward	8.26 (1.49)	8.04 (1.75)	0.370			
Ward Environment						
No night-time noise from other patients	8.29 (1.52)	8.00 (1.85)	0.670			
No night-time noise from staff	8.54 (1.40)	6.50 (1.73)	<0.001	1.35	0.56–2.14	0.001
High levels of ward cleanliness	8.48 (1.61)	7.45 (1.54)	0.005			
No threatening behaviour from other patients or visitors	8.22 (1.64)	6.50 (0.71)	0.080			
High satisfaction with the food	8.23 (1.59)	8.27 (1.40)	0.980			
Sufficient help at mealtimes	8.71 (1.11)	7.50 (2.12)	0.450			
Enough nurses on the ward	8.32 (1.34)	7.92 (2.10)	0.710			
Sufficient privacy for clinical discussions	8.55 (1.22)	6.42 (2.23)	0.001	0.09	−1.04-1.21	0.870
Sufficient privacy for examination and treatment	8.40 (1.35)	5.40 (2.61)	0.010			
Patient-staff interaction						
Confidence and trust in doctors responsible for care	8.33 (1.58)	6.20 (1.30)	0.006			
Did not experience doctors talking in front of patients as if they were not present	8.44 (1.40)	7.14 (2.14)	0.020			
Confidence and trust in nurses	8.41 (1.42)	6.14 (2.19)	0.008	1.51	0.54–2.49	0.003
Did not experience nurses talking in front of patients as if they were not present	8.21 (1.65)	7.80 (1.79)	0.520			
Staff to talk to about worries and fears	8.32 (1.51)	7.40 (2.03)	0.100			
Sufficient emotional support from staff	8.65 (1.17)	5.73 (1.84)	<0.001			
No pain	8.16 (1.80)	8.29 (1.64)	0.780			
Sufficient pain control from staff	8.42 (1.49)	6.67 (1.80)	0.004			
Information and involvement in treatment						
Important questions answered by doctors	8.47 (1.30)	5.88 (2.30)	0.002	0.83	0.18–1.84	0.100
Important questions answered by nurses	8.35 (1.47)	6.33 (2.34)	0.030			
Involvement in decisions about treatment	8.53 (1.30)	6.36 (2.11)	<0.001	0.72	−0.32-1.77	0.170
Confidence in decisions made about treatment	8.38 (1.60)	6.63 (1.19)	0.001			
Sufficient information given about treatment	8.51 (1.35)	6.45 (2.02)	<0.001	0.86	0.01–1.70	0.047
Sufficient explanation of risks and benefits of surgery	8.46(1.40)	6.70 (2.21)	0.006	1.26	0.18–2.34	0.020
Sufficient explanation of operation details	8.44 (1.45)	6.80 (2.10)	0.006			
Questions answered about surgery	8.36 (1.44)	6.33 (3.06)	0.160			
Sufficient pre-op explanation of what to expect post-op	8.52 (1.33)	7.35 (2.03)	0.020			
Sufficient explanation from anaesthetists	8.20 (1.67)	7.50 (0.71)	0.290			
Sufficient post-op explanation of operation findings	8.53 (1.39)	7.26 (1.94)	0.007			
Discharge Process						
Involvement in discharge decision-making	8.56 (1.22)	6.71 (2.23)	0.002			
Sufficient notice prior to discharge	8.49 (1.50)	7.24 (1.75)	0.004			
Discharge not delayed	8.27 (1.96)	7.95 (1.52)	0.670			
Provision of written information	8.71 (1.17)	7.31 (1.93)	0.001	−0.20	−0.86- 0.44	0.520
Explanation of purpose of discharge medication	8.42 (1.35)	5.60 (2.51)	0.008			
Explanation how to take discharge medication	8.34 (1.50)	6.25 (2.36)	0.040			
Warning of danger signals to look out for at home	8.54 (1.47)	7.63 (1.78)	0.010			
Consideration of family situation in planning discharge	8.77 (1.00)	6.77 (2.24)	0.001			
Sufficient information given to family	8.82 (1.00)	7.04 (1.99)	<0.001	0.39	−0.27-1.05	0.240
Information given for who to contact if concerned	8.52 (1.36)	6.83 (2.04)	0.003			
Discharged with required equipment/ home adaptations	8.77 (1.48)	6.00 (2.65)	0.050			
Discharged with all required community/ social care	8.33 (1.65)	6.20 (2.17)	0.040			
Overall Experience						
Treated with dignity	8.59 (1.28)	5.80 (1.55)	<0.001			
Felt well-looked after in hospital	8.64 (1.30)	6.23 (1.59)	<0.001			

#### Admission

No admission process variable was significantly associated with overall satisfaction.

#### Ward environment

The perception of sufficient privacy for both clinical discussions (8.55 (±.1.22) vs. 6.42 (±2.23), *p* = 0.001) and examinations (8.40 (±1.35) vs. 5.40 (±2.61), *p* = 0.010) was associated with increased satisfaction. The absence of night-time noise from staff (overall satisfaction score yes: 8.54 (±1.73) vs. overall satisfaction score no: 6.50 (±1.4), *p* < 0.001) and high standards of ward cleanliness (8.48 (±1.61) vs. 7.45 (±1.54), *p* = 0.005) were associated with significantly higher satisfaction scores. However, night-time noise from other patients did not have a significant effect on satisfaction (8.29 (±1.52) vs. 8.00 (±1.85), *p* = 0.670).

#### Patient-staff interaction

Confidence and trust in doctors (8.33 (±1.58) vs. 6.20 (±1.30), *p* = 0.002) and nurses (8.41 (±1.42) vs. 6.14 (±2.19), *p* = 0.008) was associated with higher overall satisfaction, as was sufficient emotional support from staff (8.65 (±1.51) vs. 5.73 (±1.84), *p* < 0.001) and good pain control (8.42 (±1.49) vs. 6.67 (±1.80), *p* = 0.010).

Indicators of good communication skills such as not talking in front of the patient without involving them in the conversation (8.44 (±1.40) vs. 7.14 (±2.14), *p* = 0.020), providing understandable and comprehensive explanations (8.51 (±1.35) vs. 6.45 (±2.02), *p* < 0.001) and answering important questions also were all associated with higher scores. Patients who felt that they were well looked after (8.64 (±1.30) vs. 6.23 (±1.50), *p* < 0.001) and treated with dignity (8.59 (±1.28) vs. 5.80 (±1.55), *p* < 0.001) during their stay were significantly more satisfied with the overall experience.

#### Information and involvement with treatment

Patients who felt that doctors had answered important questions had higher overall satisfaction scores (8.47 (±1.30) vs. 5.88 (±2.30), *p* = 0.002). This was also true for questions answered by nurses (8.35 (±1.47) vs. 6.33 (±2.34), *p* = 0.030). Patient involvement in the decision making process was also important (*p* < 0.001), with patients who felt involved giving a mean overall satisfaction score of 8.53 (±1.3), compared with 6.36 (±2.11) for those who felt uninvolved.

#### Discharge

Patients who felt that they had been involved in the decision to discharge them (8.56 (±1.22) vs. 6.71 (±2.23), *p* = 0.002), with sufficient notice (8.49 (±1.50) vs. 7.24 (±1.75), *p* = 0.004) and consideration given to their home situation (8.77 (±1.0) vs. 6.77 (±2.24), *p* = 0.001), were considerably more satisfied with their overall experience. Discharge information had a uniformly positive effect on satisfaction: instruction on warning signs to look out for at home (8.54 (±1.47) vs. 7.63 (±1.78), *p* = 0.010), the provision of written information (8.71(±1.17) vs. 7.31(±1.93), *p* = 0.001), contact information for concerns after discharge (8.52 (±1.36) vs. 6.83 (±2.04), *p* = 0.003), explanation of the discharge medication (8.42 (±1.35) vs. 5.60 (±2.51), *p* = 0.008) and instructing the family about how to care for the patient at home (8.82 (±1.00) vs. 7.04 (±1.99), *p* < 0.001) were all associated with higher satisfaction. Delayed discharge did not have an effect (8.27(±1.96) vs. 7.95 (±1.52), *p* = 0.670).

### Multivariable analysis

In a well-fitting model with an R^2^ score of 0.71, several factors were found to be independently associated with overall satisfaction scores (Table 2). “Confidence and trust in nurses” was associated with better overall satisfaction. The β-estimate was 1.51, which is interpreted as a positive response for “Confidence and trust in nurses” being associated with a 1.51 increase in overall satisfaction (95% CI 0.54–2.49, *p* = 0.003), if all other factors are held constant. Comprehensive information provision is a key contributor to satisfaction, with ‘sufficient information given about treatment’ (β = 0.86, 95% CI 0.01–1.70, *p* = 0.050) and ‘sufficient explanation of the risks and benefits of surgery’ (β = 1.26, 95% CI 0.18–2.34, *p* = 0.020) both associated with higher scores. “No night-time noise from staff” (β = 1.35, 95% CI 0.56–2.14, *p* = 0.001) also remained associated with higher score.

## Discussion

Improving patient experience is a valid endeavour in itself, and numerous studies have demonstrated an association between patient experience and improved clinical outcomes, patient safety and reduced healthcare costs [20]. That said, patient experience is a subjective and multi-factorial phenomenon, and literature concerning patient experience can be nebulous and imprecise. This study identifies two dominant themes central to good patient experience: good information provision and a pleasant and caring ward environment are consistently associated with higher overall patient satisfaction. It is too easy to say that this is obvious, yet there are many aspects of the patient pathway examined in this study that are not strongly associated with satisfaction.

The results demonstrate that patients who felt well informed about their condition and their treatment, and had received good explanations, both pre and post-operatively, reported higher levels of satisfaction with their overall experience. There is a strong body of evidence supporting information as a determinant of good patient experience in a wide variety of clinical settings, including acute care [21]. The clinical team are a prime source of patient information during the admission, and ensuring enough time to explain and discuss the diagnosis and treatment options with each patient has been identified as a key component of good clinical care [22]. It has been recognised that patients’ information requirements change during the course of an illness [23], and the realities of a busy surgical take may mean that clinical team alone may struggle to meet patients’ information requirements at all stages during their admission.

Different methods of providing information could compliment the oral information given by the clinical team. Traditional methods such as written patient information leaflets explaining the condition and treatment options and covering frequently asked questions about post-operative recovery [24] remain a simple and easy way of providing patients with supplementary information. Furthermore, the digital era has transformed the way that some patients access health information, and there has been a rapid growth in the websites and apps available to inform, educate and empower patients [25]. However, a “Digital Divide” has been recognised between the ability of different age and socioeconomic groups to access electronic resources, with the patients who stand to benefit the most from information provision the least able to access it electronically [26]. Care needs to be taken when introducing electronic resources to ensure that they do not perpetuate health inequalities, and that the information requirements of those who lack Internet access are met through other methods.

It has long been recognised that night-time noise has a deleterious effect on patient experience [27]. It is interesting to note that although patients reported experiencing night-time noise from other patients, only night-time noise from staff had a significant effect on overall patient satisfaction. Even though there must be a significant overlap in the noise from fellow patients and the noise from staff overnight, patients seem to make a distinction between the two when asked to evaluate their overall experience. This suggest that patients are willing to tolerate some aspects of a hospital admission such as noise from a distressed patient at night without it having a significant impact on their overall satisfaction, whereas other aspects, such as noise from staff, colours their perception of the entire experience. Furthermore, night-time noise highlights the dilemma around what is realistically modifiable in a hospital setting, and how to manage conflicting priorities between doing things that improve the patient experience for an individual patient (such as ensuring a quiet ward at night) with other clinical considerations (such as delivering safe care 24 h a day on a busy acute ward). The nature a busy general surgery ward makes it very difficult to completely eliminate night-time noise, however the result suggest that taking measures to reduce the noise created by staff working at night could significantly improve the overall patient experience.

The strengths of using a questionnaire-based survey to study patient experience lies in that it utilizes a validated tool that can be relatively quickly and cheaply administered to large numbers of people, generating generalizable and easily analysed data that can be tracked over time and compared with other centres [28]. However, relying on questionnaires alone may result in the collection of only superficial data, with depth and nuance lost in attempting to reduce the complexity and diversity of experiences encompassed in one patient episode into simple, closed generalizable questions [29]. To further elucidate beliefs on what constitutes a “good patient experience” would require qualitative research methods such as focus groups and semi-structured interviews to inductively explore patient expectations of their hospital stay.

At 78%, the response rate for this study is high: a typical response rate of between 63 and 69% is quoted in the literature describing the development of this questionnaire [30]. It is also considerably higher than the average 60% response rate for medical mail surveys [31]. Despite this, this study is limited by the fact it has a relatively small sample size. Undeniably, a degree of selection bias was introduced by the time limitation on following up patients after discharge, which meant that older, sicker patients with protracted inpatient stays were under represented (Fig. 1). Studies have suggested that older patients tend to report higher patient satisfaction scores [32, 33], however, longer hospital stays have been associated with lower patient satisfaction [34], therefore it is difficult to assess the likely impact of this on the data.

Traditional methods of evaluating clinical outcomes, such as complication rates, length of stay and 30-day mortality, have an important role in evaluating outcomes, but it is crucial to recognise their limitations. Traditional methods are limited in the scope of what they measure and what is consider to be a "poor outcome'. Lack of morbidity and mortality is not an adequate surrogate measure for good care. A patient may have technically perfect surgery, a prompt discharge and suffer no complications but spend their time in hospital frightened and anxious, being cared for on a dirty, noisy ward by an indifferent clinical team without any explanation or involvement in the decisions around their care. Arguably that patient has received poor care, but would have had a "good” outcome according to traditional measures. Using patient experience as an outcome measure allows for a more holistic and patient-centred evaluation of service delivery, and highlights ways of improving care in a way that matters to patients. Patient experience measures have been shown to be robust, distinctive indicators of healthcare quality [35], and have been successfully used to drive local improvement strategies across a number of healthcare settings within the NHS [36].

## Conclusion

In summary, provision of good quality information both during inpatient stay and on discharge are crucial patient experience factors associated with patient satisfaction. Improving the ward environment, particularly at night, can be challenging, but reducing noise may also improve the overall experience in emergency surgery. These findings could be utilised to inform patient experience improvement strategies, and further research is required to evaluate the impact of such strategies on overall patient experience.

## Abbreviations

GIS: General inpatient survey
MRC: Medical research council
PREMs: Patient reported experience measures
SD: Standard deviation
